# Effectiveness of Digital Cognitive Behavioural Therapy (dCBT) for Co-Occurring Anxiety, Depression and Chronic Pain in Adults: A Systematic Review and Meta-Analysis

**DOI:** 10.62641/aep.v54i4.2138

**Published:** 2026-08-15

**Authors:** Li Zhu, Yanping Ji, Juner Le, Henan Wu

**Affiliations:** ^1^Psychosomatic Department, Zhoushan Second People’s Hospital, 316000 Zhoushan, Zhejiang, China

**Keywords:** digital cognitive behavioural therapy, chronic pain, depression, anxiety, comorbidity

## Abstract

**Background:**

This study aimed to evaluate the effectiveness of digital cognitive behavioural therapy (dCBT) in reducing pain intensity, pain interference, depression, and anxiety symptoms in adults with co-occurring chronic pain, anxiety and depression.

**Methods:**

We systematically searched in PubMed, PsycINFO, Cochrane Library and Web of Science for randomized controlled trials (RCTs) published up to October 2025. Included studies comparing dCBT with control conditions in adults with chronic pain (≥3 months), co-occurring anxiety and depression. Primary outcomes were pain intensity and depressive symptoms. Secondary outcomes were pain interference and anxiety symptoms. Pooled standardized mean difference (Hedges’ g) were calculated using fixed-effects or random-effects models depending on heterogeneity.

**Results:**

We included 6 RCTs involving 737 participants with chronic pain, co-occurring anxiety and depression. Compared with control groups, dCBT did not significantly reduce pain intensity (g = –0.43, 95% confidence interval (CI) [–0.98, 0.11], *p* = 0.12; I^2^ = 91%), but it significantly reduced depressive symptoms (g = –0.51, 95% CI [–0.66, –0.36], *p* < 0.00001; I^2^ = 35%), pain interference (g = –0.70, 95% CI [–1.18, –0.21], *p* = 0.005; I^2^ = 89%), and anxiety symptoms (g = –0.59, 95% CI [–0.74, –0.44], *p* < 0.00001; I^2^ = 37%).

**Conclusions:**

dCBT effectively improves depressive symptoms, anxiety symptoms, and pain interference in patients with co-occurring chronic pain, anxiety and depression, but its direct effect on pain intensity did not reach statistical significance in this meta-analysis. dCBT is convenient and scalable, with confirmed efficacy in emotional and functional domains, making it a valuable adjunctive treatment option. Future research should investigate its true effect on pain intensity, long-term outcomes and personalized treatment approaches.

## Introduction

Chronic pain, defined as pain lasting or recurring for more than three months, is a leading cause of disability worldwide, affecting an estimated 20% of the adult population [1]. Beyond its sensory dimension, chronic pain is a complex biopsychosocial experience that frequently coexists with anxiety and depression [2]. The prevalence of depressive and anxiety disorders in patients with chronic pain is approximately 40% [3]. This co-occurrence is bidirectional and synergistic: pain exacerbates anxiety and depression, while depression and anxiety can lower pain thresholds, amplify pain perception, and impede functional recovery through mechanisms (e.g., fear-avoidance and catastrophizing) [4, 5]. Co-occurring conditions create bidirectional pathophysiological mechanisms—including amplified neuroinflammatory processes, shared genetic vulnerabilities, and reciprocal reinforcement of pain catastrophizing and negative affect—necessitating targeted intervention approaches [6]. This integrated management is supported by recent clinical guidelines emphasizing the need to address psychological distress to achieve sustainable pain reduction [7, 8].

Cognitive behavioural therapy (CBT) is the gold-standard psychological treatment for chronic pain, anxiety and depression. It targets maladaptive thoughts, emotions and behaviours that perpetuate the cycle of pain and distress [9]. However, access to face-to-face CBT is limited by marked barriers, including high costs, geographical constraints, long waiting lists, and stigma associated with mental health treatment.

Digital cognitive behavioural therapy (dCBT), delivered via web platforms or mobile applications, has emerged as a promising solution to overcome these barriers [10]. By offering structured, evidence-based content in an accessible and flexible format, dCBT has the potential to democratize access to care. Separate meta-analyses have established the efficacy of dCBT for chronic pain [11], depression and anxiety disorders [12, 13] individually. However, those reviews excluded populations with co-occurring conditions or combined heterogeneous samples, limiting conclusions about this high-need subgroup.

Despite this progress, a critical gap remains in understanding the synthesized effect of dCBT specifically for the co-occurring of chronic pain, anxiety and depression. Patients with this co-occurrence often have more severe symptoms and poorer treatment outcomes. An intervention that can simultaneously target both domains is therefore of high clinical importance. This meta-analysis aimed to fill this gap by synthesizing evidence from randomized controlled trials (RCTs) to determine the effectiveness of dCBT in reducing co-occurring pain-related outcomes and symptoms of depression and anxiety. To our knowledge, this is the first meta-analysis to specifically examine dCBT for co-occurring chronic pain, anxiety and depression, addressing a critical gap between separate pain and mental health literatures. By integrating RCT data, we provided evidence for dCBT as a scalable, integrated treatment approach.

## Methods

This meta-analysis was conducted and reported in accordance with the Preferred Reporting Items for Systematic Reviews and Meta-Analyses (PRISMA) 2020 statement (Presented as **supplementary materials**).

### Search Strategy

We systematically searched PubMed, PsycINFO, the Cochrane Central Register of Controlled Trials, and Web of Science from inception to 31 October 2025. The search strategy combined keywords and MeSH terms related to the intervention (e.g., “cognitive behavioral therapy”, “internet based intervention”), the population (e.g., “chronic pain”, “fibromyalgia”, “back pain”) and the co-occurring conditions (e.g., “depression”, “anxiety”, “emotional distress”). Reference lists of included studies and relevant reviews were also manually screened. No eligible studies were identified before 2010, consistent with the emergence of dCBT interventions during this period. The search was restricted to English-language publications; grey literature (e.g., conference abstracts, unpublished trials) was not included due to resource constraints, which may introduce language and publication bias.

### Inclusion and Exclusion Criteria

Studies were included if they met the following criteria:

(1) Participants: Adults (≥18 years) with a primary diagnosis of chronic pain (duration ≥3 months) and co-occurring clinical or elevated symptoms of anxiety and depression. Elevated symptoms were defined as scores ≥1.5 standard deviations above normative means on validated measures or clinical diagnoses confirmed via structured interviews (e.g., Patient Health Questionnaire-9 ≥10 for depression [14], Generalized Anxiety Disorder-7 ≥8 for anxiety [15], or Hospital Anxiety and Depression Scale ≥8 for combined anxiety and depression [16]).

(2) Intervention: A dCBT program, defined as a structured psychological intervention based on CBT principles delivered primarily via the internet or a mobile application. Interventions could be guided (with therapist support) or unguided.

(3) Comparator: Any control condition, including waitlist control (WLC), treatment as usual (TAU), or an active control (e.g., online education).

(4) Outcomes: At least one validated measure of pain (e.g., pain intensity, pain interference) and at least one validated measure of anxiety and depression (e.g., depression, anxiety symptoms).

(5) Study Design: Published, peer-reviewed RCTs with clearly defined intervention and control groups, reporting post-intervention outcome data. Studies focusing solely on symptom measurement, qualitative data, or non-randomized designs (e.g., cohort studies) were excluded. Narrative reviews and face-to-face CBT interventions were excluded to maintain on primary digital interventions.

### Data Extraction and Quality Assessment

Two independent reviewers screened titles, abstracts, and full texts for eligibility. Disagreements were resolved by a third reviewer. Inter-rater reliability was excellent (Cohen’s kappa = 0.86 for eligibility screening, 0.92 for data extraction). A standardized data extraction form was used to collect information on study characteristics (e.g., author, year, country), participant demographics, pain condition, intervention and comparator details and outcome measures.

The methodological quality of included RCTs was assessed using the Cochrane Risk of Bias 2 tool.

### Statistical Analysis

The prespecified primary outcomes were post-treatment assessments of pain intensity and depressive symptom severity, whereas secondary outcomes consisted of pain interference and anxiety symptom endpoints. We calculated standardized mean difference (SMD) using Hedges’ g for each outcome, which is suitable for combining results from different scales. Effect sizes were interpreted as small (g = 0.20), medium (g = 0.50), or large (g = 0.80) [17]. Heterogeneity was quantified using the I^2^ statistic, with values of 25%, 50%, and 75% considered low, moderate, and high, respectively [18]. A fixed-effects model was used for pooling when I^2^ < 50%, and a random-effects model when I^2^ ≥ 50%. All analyses were conducted using Review Manager Version 5.4 (The Cochrane Collaboration, London, UK).

## Results

### Study Selection

**Fig. 1.**
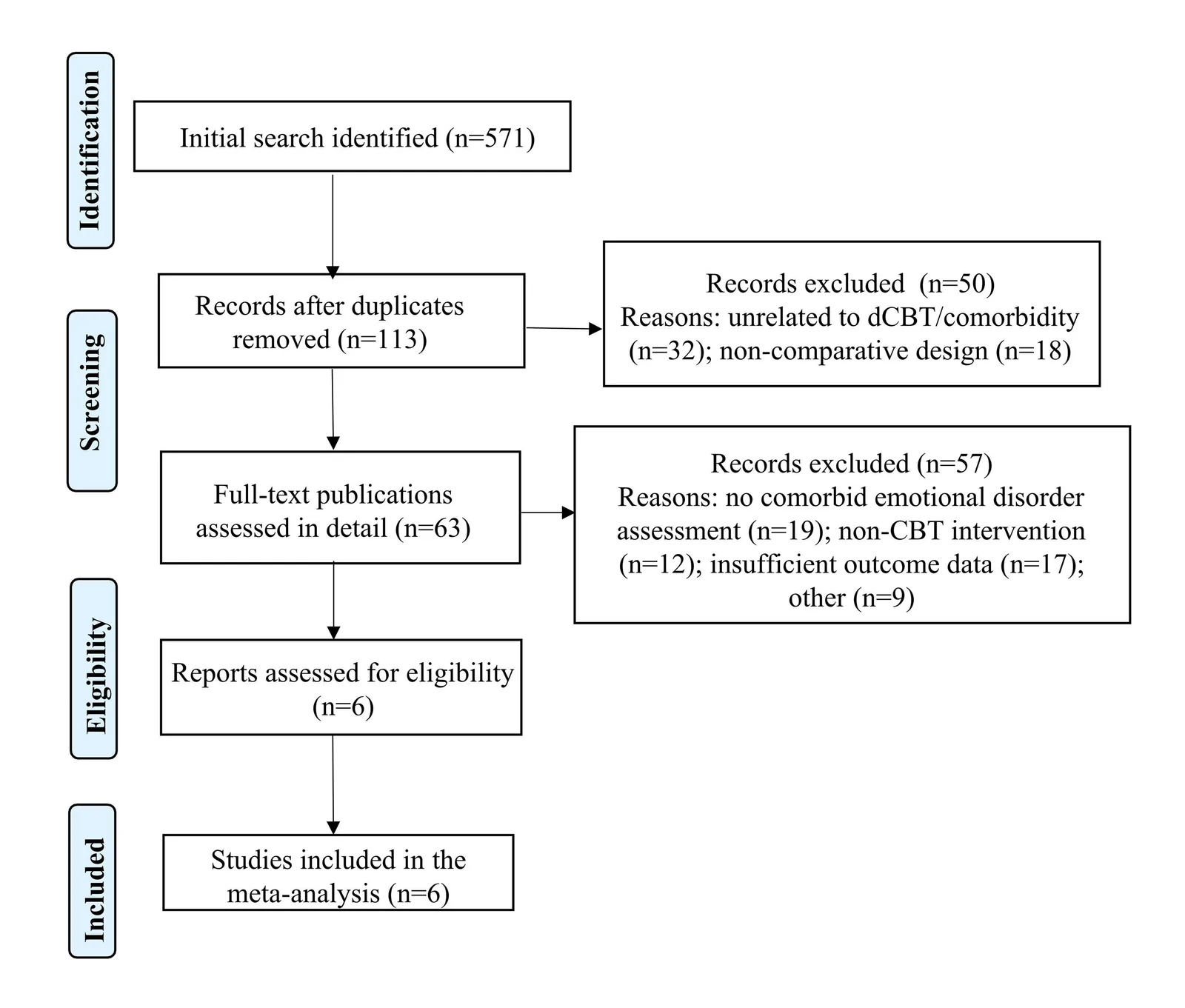
**The PRISMA flow diagram**. dCBT, digital cognitive behavioural therapy; CBT, cognitive behavioural therapy.

The initial search identified 571 records. After duplicate removal, 113 unique citations proceeded to title and abstract screening. Of these, 50 were excluded at this stage: 32 publications were irrelevant to dCBT or the target co-occurring phenotype, and 18 featured non-comparative study designs, leaving 63 publications eligible for full-text appraisal. After detailed full-text assessment, 57 studies were excluded: absent validated assessment of anxiety and depression (n = 19), non-dCBT intervention components (n = 12), inadequate extractable outcome metrics (n = 17), and failure to satisfy predefined eligibility benchmarks (n = 9). Finally, 6 RCTs involving 737 participants met all inclusion criteria and were included in the systematic review and meta-analysis. The PRISMA flow diagram details the selection process (Fig. 1).

### Study Characteristics

The 6 eligible RCTs included in this meta-analysis were published through October 2025 and involved a total of 737 participants (intervention: n = 364; control: n = 373). The most common chronic pain conditions included chronic back pain (n = 4), mixed chronic pain (n = 1) and fibromyalgia (n = 1). Most dCBT interventions (n = 5) offered therapist guidance (e.g., weekly check-ins via messaging or video calls), while 1 was unguided. Control groups included waitlist control (n = 2), treatment as usual (n = 1), online education (n = 1), and active controls such as online forums (n = 2). The intervention periods varied from 6 to 12 weeks among the eligible RCTs [median = 8 weeks, Table 1 (Ref. [19, 20, 21, 22, 23, 24])].

**Table 1.** **Characteristics of included studies**.

Author (Year)	Population/Pain condition	N (I/C)	Intervention (dCBT)	Guidance	Comparator	Duration
Dear *et al*. [19] (2013)	Mixed chronic pain	60 (30/30)	Pain course	Guided	WLC	8 weeks
Buhrman *et al*. [20] (2013)	Chronic pain	76 (38/38)	ACT-based iCBT	Guided	Forum	7 weeks
Chiauzzi *et al*. [21] (2010)	Chronic back pain	199 (95/104)	painACTION	Unguided	Education	6 weeks
Serrat *et al*. [22] (2021)	Fibromyalgia	272 (135/137)	FIBROWALK	Guided	TAU	12 weeks
Schlicker *et al*. [23] (2020)	Chronic back pain	76 (40/36)	Get.Back	Guided	WLC	9 weeks
Buhrman *et al*. [24] (2011)	Chronic back pain	54 (26/28)	iCBT	Guided	Forum	12 weeks

dCBT, digital cognitive behavioural therapy; I/C, Intervention/Control; ACT, acceptance and commitment therapy; iCBT, internet-based CBT; TAU, treatment as usual; WLC, waitlist control.

### Risk of Bias Assessment

The methodological quality of the 6 RCTs was assessed using the Cochrane Risk of Bias 2 tool (Table 2, Ref. [19, 20, 21, 22, 23, 24]). Regarding the randomization process, 5 studies [19, 20, 21, 22, 24] were rated as low risk, and 1 study [23] raised some concerns. Due to the inability to blind participants and therapists to the behavioural interventions, for deviations from intended interventions, 4 studies [19, 20, 21, 23] were rated as having some concerns, while 2 studies [22, 24] were rated as low risk. In terms of outcome measurement and selective reporting of results, all studies were rated as low risk. Overall, 2 studies [22, 24] were rated as low risk of bias, and the remaining 4 studies were rated as having some concerns.

**Table 2.** **Risk of bias summary for RCTs**.

Study (Year)	D1	D2	D3	D4	D5	Overall
Dear *et al*. [19] (2013)	L	S	L	L	L	Some concerns
Buhrman *et al*. [20] (2013)	L	S	L	L	L	Some concerns
Chiauzzi *et al*. [21] (2010)	L	S	S	L	L	Some concerns
Serrat *et al*. [22] (2021)	L	L	L	L	L	Low risk
Schlicker *et al*. [23] (2020)	S	S	L	L	L	Some concerns
Buhrman *et al*. [24] (2011)	L	L	L	L	L	Low risk

D1: Bias arising from the randomization process; D2: Bias due to deviations from intended interventions; D3: Bias due to missing outcome data; D4: Bias in measurement of the outcome; D5: Bias in selection of the reported result; L: Low risk; S: Some concerns.

### Meta-Analysis Results

#### Pain Intensity

**Fig. 2.**
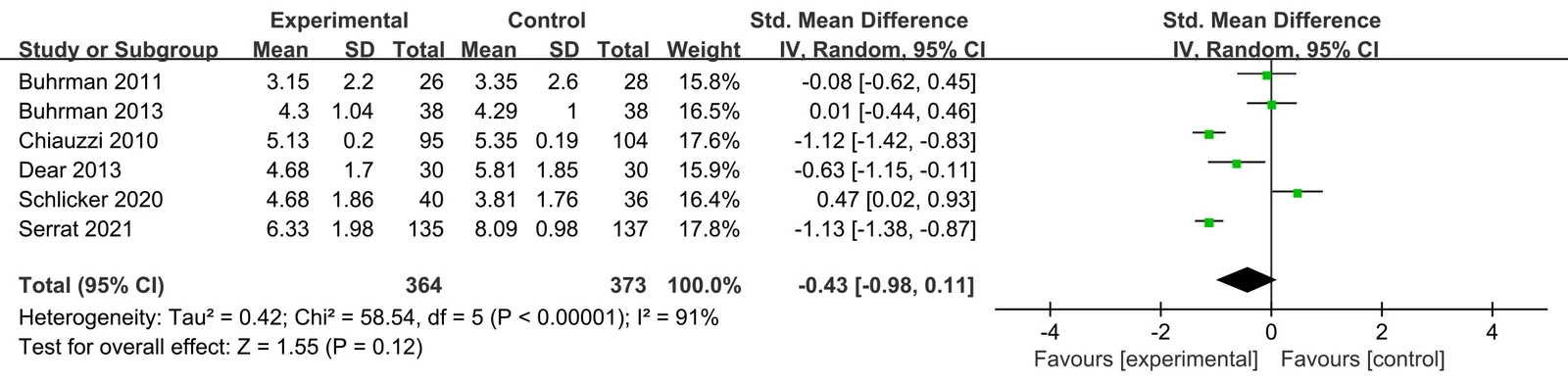
**Forest plot for pain intensity**. CI, confidence interval; SD, standard deviation.

A total of 6 studies were included in the meta-analysis for pain intensity, and a random-effects model was used for pooling due to the high heterogeneity (I^2^ = 91%, Tau^2^ = 0.42, Chi^2^ = 58.54, df = 5, *p* < 0.00001). Compared with control groups, the pooled effect size of dCBT on pain intensity was g = –0.43 (95% confidence interval (CI) [–0.98, 0.11]), indicating a small-to-medium trend toward improvement, but the difference was not statistically significant (Z = 1.55, *p* = 0.12, Fig. 2).

#### Depressive Symptoms

**Fig. 3.**
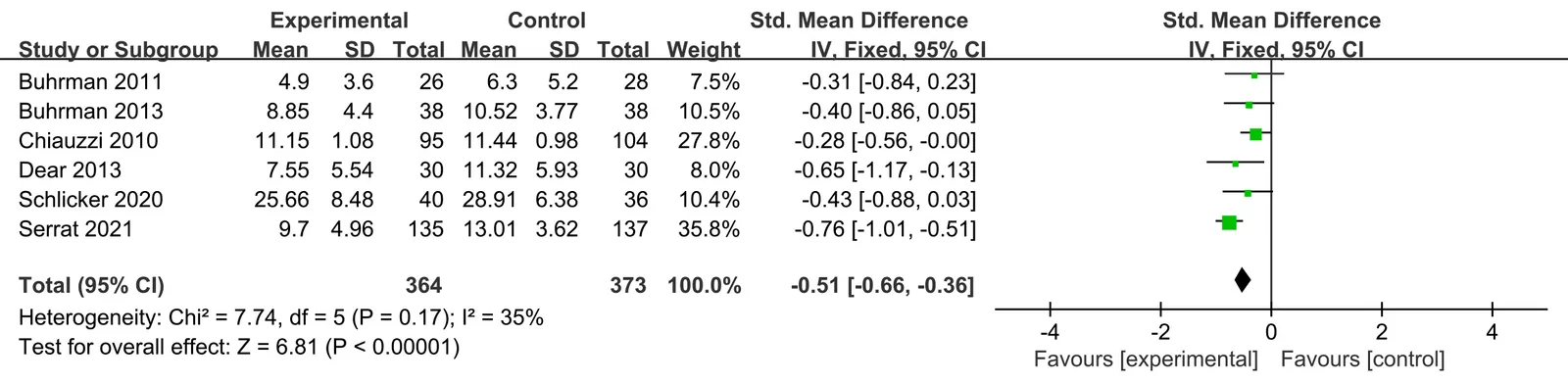
**Forest plot for depressive symptoms**. CI, confidence interval; SD, standard deviation.

A total of 6 studies were included in the meta-analysis for depressive symptoms, and a fixed-effects model was used for pooling due to the low heterogeneity (I^2^ = 35%, Tau^2^ = 0, Chi^2^ = 7.74, df = 5, *p *= 0.17). Compared with control groups, the pooled effect size of dCBT on depressive symptoms was g = –0.51 (95% CI [–0.66, –0.36]), indicating a moderate improvement, and the difference was statistically significant (Z = 6.81, *p* < 0.00001, Fig. 3).

#### Pain Interference

**Fig. 4.**
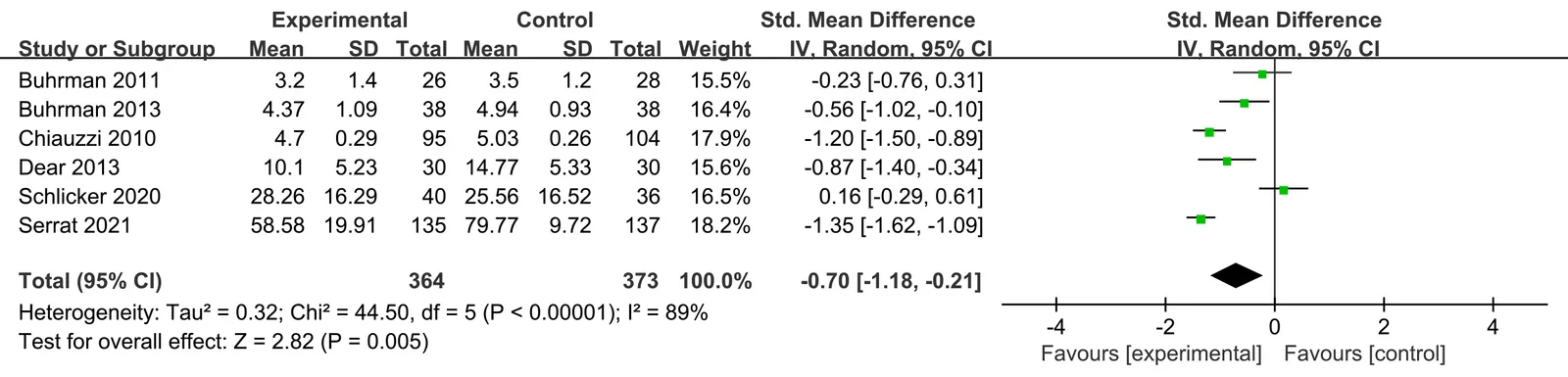
**Forest plot for pain interference**. CI, confidence interval; SD, standard deviation.

A total of 6 studies were included in the meta-analysis for pain interference, and a random-effects model was used for pooling due to the high heterogeneity (I^2^ = 89%, Tau^2^ = 0.32, Chi^2^ = 44.50, df = 5, *p* < 0.00001). Compared with control groups, the pooled effect size of dCBT on pain interference was g = –0.70 (95% CI [–1.18, –0.21]), indicating a moderate improvement, and the difference was statistically significant (Z = 2.82, *p* = 0.005, Fig. 4).

#### Anxiety Symptoms

**Fig. 5.**
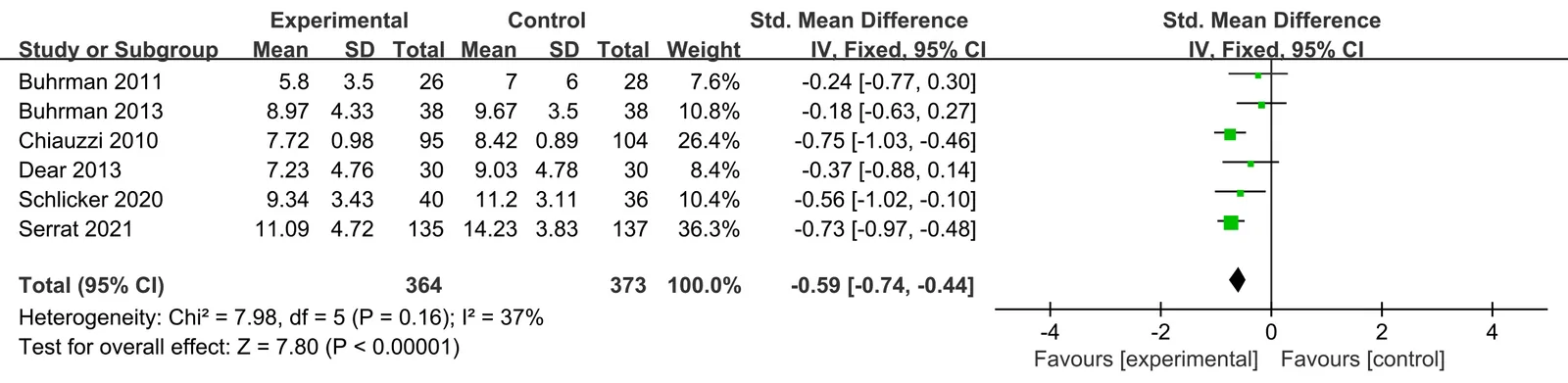
**Forest plot for anxiety symptoms**. CI, confidence interval; SD, standard deviation.

A total of 6 studies were included in the meta-analysis for anxiety symptoms, and a fixed-effects model was used for pooling due to the low heterogeneity (I^2^ = 37%, Tau^2^ = 0, Chi^2^ = 7.98, df = 5, *p *= 0.16). Compared with control groups, the pooled effect size of dCBT on anxiety symptoms was g = –0.59 (95% CI [–0.74, –0.44]), indicating a moderate improvement, and the difference was statistically significant (Z = 7.80, *p* < 0.00001, Fig. 5).

### Publication Bias

**Fig. 6.**
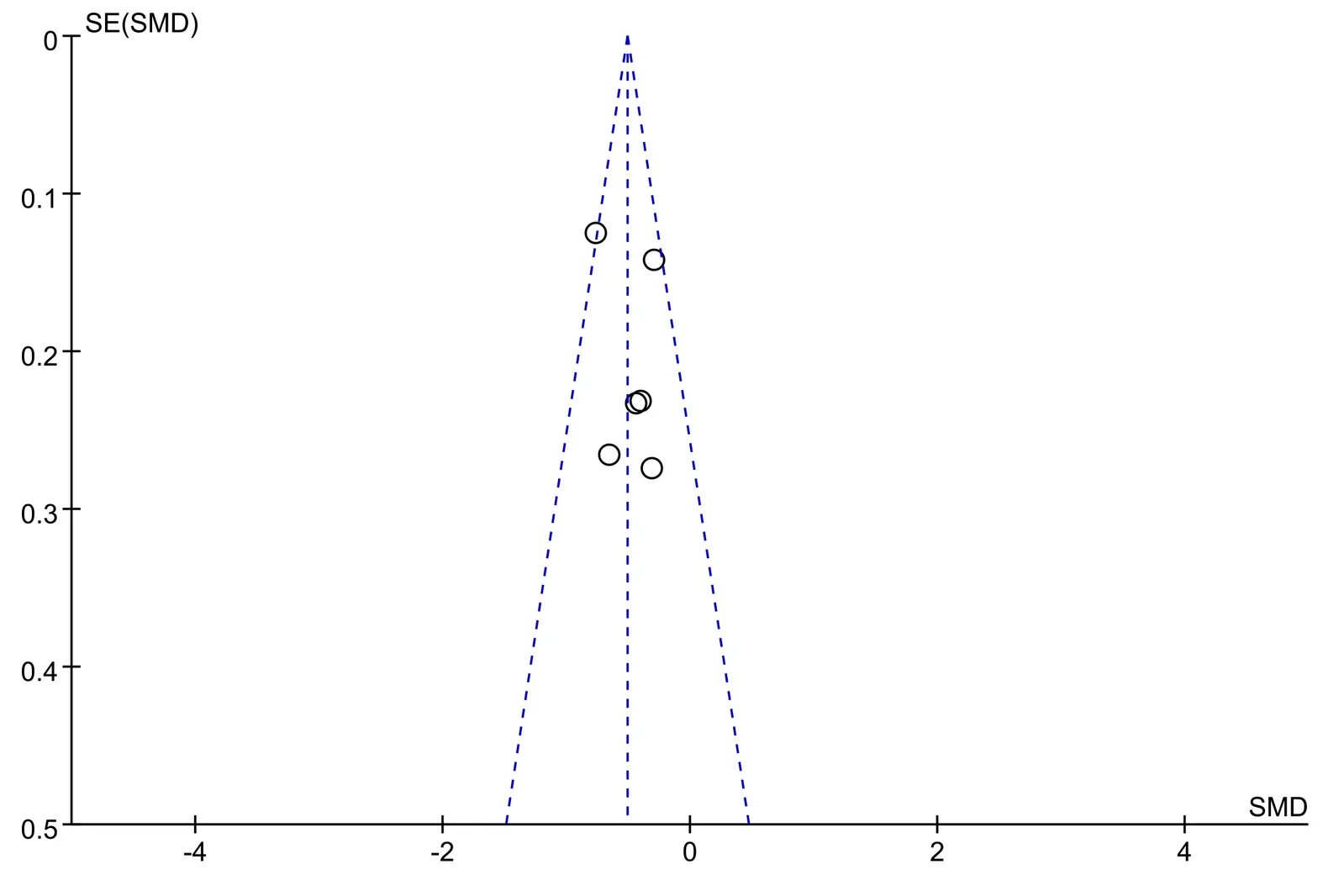
**Funnel plot for depression**. SE, standard error; SMD, standardized mean difference. Funnel plot of standard error by Hedges’ g for depression. Each dot represents one of the 6 included studies. The vertical line indicates the pooled effect size. With fewer than 10 studies, tests for funnel plot asymmetry (e.g., Egger’s test) have low power; therefore, this plot should not be used to conclude the absence of publication bias.

Publication bias was assessed using Egger’s and Begg’s tests for each of the four outcomes (Table 3). Because each meta-analysis included only 6 studies, these statistical tests have limited power; therefore, the results should be interpreted with caution, and no definitive conclusion regarding the absence of publication bias can be drawn. A funnel plot for the primary outcome (depressive symptoms) is presented as an example (Fig. 6). For the remaining outcomes, funnel plots are not shown individually because, with fewer than ten studies, visual asymmetry tests are unreliable and would not provide meaningful additional information.

**Table 3.** **Publication bias test results**.

Outcome	Egger’s test (*p*-value)	Begg’s test (*p*-value)	Interpretation (interpret with caution due to small number of studies)
Pain intensity	0.12	0.15	Non-significant, but test underpowered (k = 6)
Depression	0.09	0.11	Non-significant, but test underpowered (k = 6)
Pain interference	0.18	0.20	Non-significant, but test underpowered (k = 6)
Anxiety	0.14	0.16	Non-significant, but test underpowered (k = 6)

With only 6 studies per outcome, Egger’s and Begg’s tests have low statistical power. The *p*-values are provided for completeness but should not be interpreted as evidence for or against publication bias.

## Discussion

This systematic review and meta-analysis provides comprehensive evidence for the effectiveness of dCBT in adults with co-occurring chronic pain, anxiety and depression. These findings showed that dCBT did not significantly reduce pain intensity (g = –0.43, *p* = 0.12), but it significantly improved depressive symptoms (g = –0.51, *p* < 0.00001), anxiety symptoms (g = –0.59, *p* < 0.00001), and pain interference (g = –0.70, *p* = 0.005), with moderate effect sizes. These findings align with prior analyses demonstrating the efficacy of digital interventions for chronic pain [25, 26, 27], but extend them by specifically focusing on co-occurring anxiety and depression—a critical gap addressed by few previous reviews. Notably, in this population with co-occurring conditions, dCBT showed clear benefits on emotional and functional dimensions (pain interference), whereas the direct improvement on the sensory dimension of pain intensity did not reach statistical significance. These results underscore the promise of dCBT as an accessible component of holistic pain management, but suggest that in the context of co-occurrence, the effects of dCBT may be more evident in cognitive-emotional regulation and functional recovery than in direct analgesia.

For the significant outcomes, effect sizes (depression g = –0.51, anxiety g = –0.59, pain interference g = –0.70) are consistent with benchmarks for clinical significance in chronic pain research and comparable to those of face-to-face CBT (median effect size = 0.5) [28]. Notably, the effect size for pain interference reached a moderate-to-large level (g = –0.70), exceeding the minimal clinically important difference threshold (approximately 0.5 standard deviation) for patient-reported pain interference improvement, indicating a clinically meaningful enhancement in functional abilities [29]. For the non-significant pain intensity outcome, although g = –0.43 did not reach statistical significance, its point estimate was close to a small-to-medium effect, suggesting possible Type II error or limitations due to sample size and heterogeneity of the included studies.

Regarding heterogeneity, fixed-effects models were used for depression and anxiety outcomes (I^2^ < 50%), while random-effects models were used for pain intensity and pain interference (I^2^ ≥ 50%), indicating high level of heterogeneity for the latter two outcomes. Potential contributors include variations in target pain conditions (e.g., fibromyalgia vs. back pain), intervention components (e.g., specific CBT techniques, inclusion of acceptance and commitment therapy elements, mindfulness), level of therapist guidance (guided vs. unguided), program duration (6–12 weeks), and the nature of the control groups (waitlist vs. active control). The non-significant pooled result for pain intensity, along with its high heterogeneity, may be related to differences in pain scales used across studies and their assessment time windows. Consequently, the pooled estimates for pain outcomes should be interpreted with considerable caution. Future subgroup analyses with larger numbers of studies are needed to disentangle these effects and identify optimal intervention characteristics and patient profiles. The focus on the highly prevalent and burdensome co-occurrence of chronic pain, anxiety and depression addresses a critical clinical need.

This study has several limitations. Firstly, although we included 2 studies rated as low risk of bias and 4 studies with some concerns, the relatively small number of studies precluded robust subgroup analyses to explore specific moderators such as pain type or platform design. The four studies with “some concerns” under Cochrane RoB 2.0 indicate potential bias in the domain of deviations from intended interventions, which may affect the reliability of the pooled estimates. Secondly, because each meta-analysis included only 6 studies, the publication bias analyses (Egger’s and Begg’s tests, funnel plot) have low statistical power. Therefore, the non-significant p-values do not rule out the presence of publication bias, and the funnel plot should be interpreted with caution. Thirdly, there was moderate-to-high heterogeneity for outcomes such as pain intensity and pain interference, likely due to variations in intervention duration and levels of therapist support. Fourthly, although the included studies reported follow-up data for the intervention group at 3 or 6 months, these data were derived from within-group pre-post comparisons and lacked concurrent control group data, thus preventing their inclusion in the meta-analysis. Moreover, none of the studies reported long-term follow-up data beyond one year, which limits our ability to evaluate the sustained long-term effects of dCBT in the population with co-occurring anxiety and depression. Fifthly, the non-significant result for pain intensity may also be related to the limited sample size, suggesting that future larger-scale RCTs are needed to confirm the true effect of dCBT on pain intensity.

Clinically, although dCBT did not significantly reduce pain intensity, it demonstrated clear and clinically relevant benefits in reducing depression, anxiety, and pain interference with daily life. Therefore, these findings strongly support the integration of dCBT into routine care pathways for individuals with co-occurring chronic pain, anxiety and depression. Its digital format enables broad accessibility, positioning it as a valuable tool for bridging treatment gaps, shortening waiting lists, and delivering care in geographically remote regions. For patients with co-occurring anxiety and depression or pain-related functional limitations, dCBT is particularly recommended as an initial intervention or adjunct in a stepped-care model.

Future research should prioritize longitudinal effectiveness studies with extended follow-up periods (≥6 months) to assess the durability of dCBT effects, particularly regarding the maintenance of co-occurring symptoms. Larger-scale RCTs are especially needed to clarify the true effect of dCBT on pain intensity, as the current meta-analysis showed a medium effect size trend that did not reach statistical significance, possibly due to insufficient statistical power. Additionally, head-to-head comparative trials are needed to directly compare dCBT with face-to-face CBT and alternative digital interventions (e.g., mindfulness-based programs) among patients with co-occurring conditions. Personalized intervention research should investigate moderators of treatment response, including baseline symptom severity, co-occurrence profiles, and digital literacy, to identify optimal candidates for dCBT. Methodological advancements, including standardized outcome measures and reporting of intervention protocols, are essential to reduce heterogeneity in meta-analyses. Cost-effectiveness analyses comparing guided versus unguided dCBT models will inform resource allocation and healthcare policy. Adolescent-specific studies with adequate sample sizes are warranted to evaluate dCBT efficacy in this understudied population, with considering developmental adaptations to intervention content. Finally, mechanistic studies exploring mediators of change (e.g., catastrophizing, fear-avoidance) will help refine dCBT components for co-occurring pain, anxiety and depression, particularly in understanding why dCBT improves pain interference more effectively than pain intensity.

## Conclusions

dCBT is an effective and accessible intervention for adults with co-occurring chronic pain, anxiety and depression. It demonstrates clear benefits in reducing depressive symptoms, anxiety symptoms and pain interference, but the direct effect on pain intensity did not reach statistical significance in this meta-analysis. As healthcare systems increasingly embrace digital solutions, dCBT stands out as a promising and evidence-based approach, particularly suitable for improving anxiety, depression and functional limitations in patients with co-occurring conditions, thereby providing integrated care for this complex patient population. Future large-scale RCTs are needed to clarify the true effect of dCBT on pain intensity and to optimize intervention protocols to enhance its analgesic effects.

## Availability of Data and Materials

The datasets used and/or analysed during the current study were available from the corresponding author on reasonable request.

## Supplementary Material

Supplementary material associated with this article can be found, in the online version, at https://doi.org/10.62641/aep.v54i4.2138.

## References

[1] Shetty A, Delanerolle G, Cavalini H, Deng C, Yang X, Boyd A (2024). A systematic review and network meta-analysis of pharmaceutical interventions used to manage chronic pain. *Scientific Reports*.

[2] Miller-Matero LR, Scherrer JF, Ballantyne JC (2024). Depression and Pain. *Pain, the Opioid Epidemic, and Depression*.

[3] Aaron RV, Ravyts SG, Carnahan ND, Bhattiprolu K, Harte N, McCaulley CC (2025). Prevalence of Depression and Anxiety Among Adults With Chronic Pain: A Systematic Review and Meta-Analysis. *JAMA Network Open*.

[4] Ma M, Zhang Y, Tao K, Lu Z (2025). Neurochemical crossroads: exploring the neurotransmitter network in chronic pain and depression comorbidity. *Frontiers in Molecular Neuroscience*.

[5] Liu G, Liu D, Shi D, Wang Z, Fu W (2025). Electroacupuncture Ameliorates Chronic Inflammatory Pain and Depression Comorbidity by Inhibiting Nrf2-Mediated Ferroptosis in Hippocampal Neurons. *Neurochemical Research*.

[6] Luo K, Zhang M, Tu Q, Li J, Wang Y, Wan S (2025). From gut inflammation to psychiatric comorbidity: mechanisms and therapies for anxiety and depression in inflammatory bowel disease. *Journal of Neuroinflammation*.

[7] Wie C, Dunn T, Sperry J, Strand N, Dawodu A, Freeman J (2025). Cognitive Behavioral Therapy and Biofeedback. *Current Pain and Headache Reports*.

[8] Cojocaru CM, Popa CO, Schenk A, Suciu BA, Szasz S (2024). Cognitive-behavioral therapy and acceptance and commitment therapy for anxiety and depression in patients with fibromyalgia: a systematic review and meta-analysis. *Medicine and Pharmacy Reports*.

[9] Resmark G, Herpertz S, de Zwaan M, Zipfel S (2024). Cognitive Behavioral Therapy Models. *Handbook of Eating Disorders and Obesity*.

[10] Brückner HA, Ell J, Kalon L, Strahler J, Ducki A, Riemann D (2025). Effectiveness of digital cognitive behavioral therapy for insomnia in nurses with shift work sleep disorder: Results of a randomized controlled trial. *International Journal of Nursing Studies*.

[11] Gandy M, Pang STY, Scott AJ, Heriseanu AI, Bisby MA, Dudeney J (2022). Internet-delivered cognitive and behavioural based interventions for adults with chronic pain: a systematic review and meta-analysis of randomized controlled trials. *Pain*.

[12] Karyotaki E, Efthimiou O, Miguel C, Bermpohl FMG, Furukawa TA, Cuijpers P (2021). Internet-Based Cognitive Behavioral Therapy for Depression: A Systematic Review and Individual Patient Data Network Meta-analysis. *JAMA Psychiatry*.

[13] Etzelmueller A, Vis C, Karyotaki E, Baumeister H, Titov N, Berking M (2020). Effects of Internet-Based Cognitive Behavioral Therapy in Routine Care for Adults in Treatment for Depression and Anxiety: Systematic Review and Meta-Analysis. *Journal of Medical Internet Research*.

[14] Kroenke K, Spitzer RL, Williams JB (2001). The PHQ-9: validity of a brief depression severity measure. *Journal of General Internal Medicine*.

[15] Grapp M, Bugaj TJ, Terhoeven V, Friederich HC, Maatouk I (2025). Screening for anxiety in patients with cancer: Diagnostic accuracy of GAD-7 items considering lowered GAD-7 cut-offs. *PLoS One*.

[16] Zigmond AS, Snaith RP (1983). The hospital anxiety and depression scale. *Acta Psychiatrica Scandinavica*.

[17] Andrade C (2020). Mean Difference, Standardized Mean Difference (SMD), and Their Use in Meta-Analysis: As Simple as It Gets. *The Journal of Clinical Psychiatry*.

[18] Higgins JP, Thompson SG, Deeks JJ, Altman DG (2003). Measuring inconsistency in meta-analyses. *BMJ: British Medical Journal/British Medical Association*.

[19] Dear BF, Titov N, Perry KN, Johnston L, Wootton BM, Terides MD (2013). The Pain Course: a randomised controlled trial of a clinician-guided Internet-delivered cognitive behaviour therapy program for managing chronic pain and emotional well-being. *Pain*.

[20] Buhrman M, Skoglund A, Husell J, Bergström K, Gordh T, Hursti T (2013). Guided internet-delivered acceptance and commitment therapy for chronic pain patients: a randomized controlled trial. *Behaviour Research and Therapy*.

[21] Chiauzzi E, Pujol LA, Wood M, Bond K, Black R, Yiu E (2010). painACTION-back pain: a self-management website for people with chronic back pain. *Pain Medicine: The Official Journal of the American Academy of Pain Medicine*.

[22] Serrat M, Sanabria-Mazo JP, Almirall M, Musté M, Feliu-Soler A, Méndez-Ulrich JL (2021). Effectiveness of a Multicomponent Treatment Based on Pain Neuroscience Education, Therapeutic Exercise, Cognitive Behavioral Therapy, and Mindfulness in Patients With Fibromyalgia (FIBROWALK Study): A Randomized Controlled Trial. *Physical Therapy*.

[23] Schlicker S, Baumeister H, Buntrock C, Sander L, Paganini S, Lin J (2020). A Web- and Mobile-Based Intervention for Comorbid, Recurrent Depression in Patients With Chronic Back Pain on Sick Leave (Get.Back): Pilot Randomized Controlled Trial on Feasibility, User Satisfaction, and Effectiveness. *JMIR Mental Health*.

[24] Buhrman M, Nilsson-Ihrfeldt E, Jannert M, Ström L, Andersson G (2011). Guided internet-based cognitive behavioural treatment for chronic back pain reduces pain catastrophizing: a randomized controlled trial. *Journal of Rehabilitation Medicine*.

[25] Li R, Srinakarin K, Vega R, Murray CB, Palermo TM (2025). Treatment expectations and pain-related outcomes in clinical trials of digital cognitive-behavioral therapy for youth with chronic pain. *The Journal of Pain*.

[26] Palermo TM, Srinakarin K, Zhou C, Lalloo C, Dampier C, Zempsky WT (2025). Moderators of digital cognitive-behavioral therapy for youth with sickle cell disease pain: secondary analysis of a randomized controlled trial. *Pain*.

[27] Schuffelen J, Maurer LF, Gieselmann A (2026). The Effects of Digital Cognitive Behavioral Therapy for Insomnia in Chronic Pain: A Randomized Controlled Trial. *Psychotherapy and Psychosomatics*.

[28] Morley S, Eccleston C, Williams A (1999). Systematic review and meta-analysis of randomized controlled trials of cognitive behaviour therapy and behaviour therapy for chronic pain in adults, excluding headache. *Pain*.

[29] Chen CX, Kroenke K, Stump TE, Kean J, Carpenter JS, Krebs EE (2018). Estimating Minimally Important Differences for the PROMIS Pain Interference Scales: Results From 3 Randomized Clinical Trials. *Pain*.

